# Early readmission and its predictors among patients treated for acute exacerbations of chronic obstructive respiratory disease in Ethiopia: A prospective cohort study

**DOI:** 10.1371/journal.pone.0239665

**Published:** 2020-10-06

**Authors:** Zenebe Kano Anbesse, Teshale Ayele Mega, Behailu Terefe Tesfaye, Getandale Zeleke Negera

**Affiliations:** 1 Clinical Pharmacy Unit, School of Pharmacy, College of Health and Medical Sciences, Haramaya University, Harar, Ethiopia; 2 Department of Clinical Pharmacy, School of Pharmacy, Institute of Health, Jimma University, Jimma, Ethiopia; BronxCare Health System, Affiliated with Icahn School of Medicine at Mount Sinai, NY, USA, UNITED STATES

## Abstract

**Background:**

Significant numbers of chronic obstructive respiratory disease patients are readmitted for Acute Exacerbation (AE) within 30 days of discharge. And these early readmissions have serious clinical and socioeconomic consequences. The objective of our study was to determine the rate of readmission within 30 days of discharge and it’s predictors among patients treated for acute exacerbations of asthma and chronic obstructive pulmonary disease (COPD).

**Methods:**

A prospective cohort study involving 130 patients (asthma = 59, COPD = 71) was conducted from April-September, 2019, in Jimma Medical Center (JMC), South-West Ethiopia. Socio-demographic, clinical, laboratory, and drug-related data were recorded at admission and during hospital stay. Cox regression analysis was performed to identify risk factors for readmissions following an AE of asthma and COPD.

**Results:**

During the study period, 130 (male, 78(60%)) patients were admitted with AE of asthma and COPD. The median age was 59(IQR, 50–70) years. Of 130 patients, 21(18.10%) had a new AE of asthma and COPD that required hospitalization in the 30 days after discharge. The overall median survival time to 30-day readmission was 20 days (IQR, 16–29). Multivariate analysis revealed prolonged use of oxygen therapy (AHR = 4.972, 95% CI [1.041–23.736] and frequent hospital admissions (AHR = 11.482 [1.308–100.793]) to be independent risk factors for early readmissions.

**Conclusion:**

Early hospital readmission rates for AE of asthma and COPD were alarmingly high. Frequent hospital admission and long-term oxygen therapy during hospital stay were independent predictors of 30-day readmission.

## Background

Asthma and Chronic Obstructive Pulmonary Disease (COPD), commonly called chronic respiratory diseases, are common diseases with a heterogeneous distribution worldwide. The clinical course is punctuated by intermittent episodes of acute worsening of symptoms, termed exacerbations [1]. Exacerbations of asthma and COPD are defined as an acute onset and worsening of respiratory symptoms beyond the baseline level that require a change in medication for mild cases and emergency department (ED) visits or hospitalization in more severe cases [2]. Exacerbations due to lack of early prevention of the risk factor and co-morbidity are the main cause of hospital admission [1, 3–5].

According to the Global Burden ofDisease (GBD) report in 2015, asthma and COPD affected an estimated 358.2 and 174.5 million people, respectively [1, 3–5]. In the same year, COPD was responsible for 3.2 million deaths and 0.4 million deaths from asthma. COPD alone is accounted for 2.6% disability adjusted life years (DALYs), while 1.1% of the DALYs belonged to asthma [1]. Similarly, COPD is attributed to nearly 800,000 hospitalizations and estimated $50 billion in healthcare expenditures, while the healthcare expenditure due to asthma was $56 billion [3, 5].

In Africa, a spirometry based study showed the prevalence of COPD to be 13.4%. But, it was 4% by non-spirometry study [6], and that of asthma was 12.8% [7]. In Ethiopia, the prevalence of asthma was 29.6% [7], while COPD was 17.8% [6]. Asthma is responsible for 0.6% of death, while death attributed to COPD ranges from 3% to 5.2% [8].

Acute exacerbations of chronic obstructive respiratory diseases like asthma may result in excess medication use, emergency department visits, hospitalization, and even death. It also reduces quality of life and leads to time off work and school with the associated emotional and financial stress which accounts for almost 50% of total costs [9]. Similarly, acute exacerbations of COPD (AECOPD) may result in morbidity, and mortality as well as loss of lung function and impaired health status [10–12]. Prevention or amelioration of exacerbations has become a major therapeutic outcome as it is a common event with a median of 1.2 to 2.4 exacerbations per patient per year [13], and the frequency of exacerbations increases with increased severity of the disease [14–16].

Furthermore, unplanned 30 days hospital readmissions are a major health care burden accounting for as much as 58% of total costs in Medicare insured populations [17]. Nearly 20% of all Medicare discharges are readmitted within 30 days and among all discharge diagnoses, asthma and COPD are major causes [18]. Although studies are scarce in resource limited settings, risk-prediction model was developed focusing on which co-morbidities and risk factors affect 30-day readmission rates for asthma and COPD. Factors like tobacco use, diabetes mellitus, infections, and obesity, frequent hospital visits, ageare some of them [19, 20]. Readmissions in asthma and COPD patients have serious clinical and socioeconomic consequences [21]. A recent study showed that up to 20% of COPD patients readmitted due to AECOPD within 30days after discharge [22].

To date, there is no study evaluating the rate of readmission within 30 days of discharge among asthma and COPD patients in Ethiopia. The aim of this study was therefore to determine the rate of readmission within 30 days of discharge and it’s predictors among asthma and COPD patients presenting with acute exacerbations.

## Methods

### Study design and setting

This prospective cohort study was conducted at a University hospital in Ethiopia (Jimma Medical Center (JMC)) over a period of 6 months starting from April-September, 2019. JMC is located in Jimma town, 355 km from Addis Ababa, capital city. It is one of the oldest public hospitals found in the South -Western part of the country that runs under Jimma University. It is currently the only medical center in this part of the country. This medical center gives a service for a catchment’s population of 15 million and has more than 800 beds, 1600 staff members. It has several specialty clinics. As one of the specialty clinics, chest clinic has outpatient department (OPD) and pulmonology unit, which was the major source of patients for this study.

### Study population and variables

We included all adult (age>18 years) patients with acute exacerbations of asthma and COPD admitted to medical wards, ED, and ICU of JMC during the study period. Cardiac asthma, Asthma-COPD overlap, asthma or COPD misdiagnosed patients and patients unwilling to participate in the study were excluded.

Data were collected on patient-related factors (socio-demographic, body mass index (BMI)), social drug use (smoking status), medication adherence, living with pet animals), disease-related factors (laboratory findings, clinical presentation, severity ofasthma and COPD at admission, duration of AE of asthma and COPD, and co morbidities), and drug related factors (in-hospital medications, past medication history, medications at discharge). The lung function test (Forced expiratory volume (FEV_1_), Forced vital capacity (FVC), Peak expiratory flow rate (PEFR), and FEV_1_/FVC) was performed by spirometryV1.34:CAR36ML3500SR4 (brand: carefusion-microlab3500 spirometry). Laboratory results of the first blood test in index admission (hemoglobin, white blood cell count (WBC), creatinine, blood urea nitrogen (BUN)) were collected. Complete blood counts (CBC) were measured using hematologic analyzers: XT-1800i (Sysmex, Japan), KX-21 N™ (Sysmex, Japan), and Cell-Dyn 18001(Abbot, USA). Blood tests including serum creatinine (Cr), blood urea nitrogen (BUN) were analyzed using chemistry analyzers: ABX Pentra 400(Horiba, USA), Dirui DR-7000D (DIRUI, Changchun, China) and HumaLyzer 3000 (HUMAN, Wiesbaden, Germany). The severity of asthma or COPD disease was evaluated using the global initiative for asthma (GINA 2019) and global obstructive lung disease (GOLD 2019) classification, respectively [23, 24]. Adherence to asthma and COPD medications inhalation techniques were assessed by passive observation of patient’s ability to demonstrate the10 steps of inhalation technique, the validated questionnaire widely used in chronic obstructive diseases.

### Outcome measurement and method of validations

Both Spirometry measurements and inhalation techniques were assessed after patients became clinically stable without respiratory distress. Before spirometry measurements, patients were asked to omit short-acting inhaled bronchodilators for 4–6 hours, and long-acting oral and inhaled agents for 12 hours. Spirometry was done initially then Salbutamol 400mcg inhaler administered in four separate doses and spirometry was repeated after 15minutes [24].

Patients were diagnosed as AECOPD, if the post bronchodilator (400mcg of SABA in our setting) peak flow measurement after 15 minutes was increased by less than 12% or 200ml from baseline of PEF or FEV_1_ (pre-bronchodilator) or evaluated FEV_1_ was less than or equal to 80% predicted from age, height, and sex, and FEV_1_/FVC ratio was less than 0.7, plus clinical feature of dyspnea, chronic cough and sputum productions [24].

Patients were diagnosed as AE of asthma if the post bronchodilator (400mcg of SABA) peak flow measurement after 15 minutes was increased by 12% or 200ml from baseline of PEF or FEV_1_ (pre-bronchodilator) and FEV_1_/FVC ratio was less than 0.7 and oxygen saturation≥90, plus clinical feature of cough, wheezing shortness of breath and presence of risk factors [25]. Finally, 30-day readmission information was obtained when readmitted to the hospital or through telephone from the family or the patient itself.

### Sample size determination and sampling technique

Since the number of patients encountered during the study period was very limited, we included all patients who fulfilled the eligibility criteria and there is no special technique employed.

### Data collection tool and procedure

The data collection tool was developed based on previous similar studies [11, 14, 16, 26–30] and active patient follow-up chart. Data were collected both from active patient follow-up chart and patient interview.

### Data quality assurance

A carefully designed data collection tool was used to collect important data required to meet the stated objectives. Prior to the actual data collection process, pre-test was conducted on 10 randomly selected eligible patients and the data collection tool was modified accordingly. Four data collectors (two pharmacists with Bachelor of Pharmacy degree and two clinical nurses with Bachelor of Science degree) and four supervisors (general practitioners) were hired and two-day training on the data collection tool and general procedures was provided by the principal investigator. The supervisors coordinate data collectors and facilitate the daily activities. All filled checklists were reviewed for completeness and consistency on a daily basis by supervisors and principal investigator.

### Statistical analysis

Data were entered into Epi-Data 4.02.01 for cleaning and exported to STATA 14 for analysis. Descriptive analysis was performed and results were presented by text, tables and figures. Survival estimates for 30-day readmission was checked by Kaplan-Meier (log-rank test). Multicolinearity test was performed to check for co-linearity between independent variables.

X^2^ test was performed to check adequacy of cells before performing Cox regression. Cox regression model assumption of proportional hazards was checked by testing an interaction of covariates with time. Bivariate Cox regression was performed to identify candidate variables for multivariable Cox regressions. Variables with p-value < 0.25 in bivariate regression were considered as candidates for multivariable Cox regression. Multivariable Cox regression was performed to identify independent predictors of 30-day readmission. Hazard ratio was used as a measure of strength of associationand p-value < 0.05 was considered todeclare statistical significance.

### Ethical approval and consent to participate

The study was approved by the Institutional Review Board (IRB) of Jimma University. It has designated with an IRB number of IHRPGD/549/109. After informing the overall concern of the study and confidentiality of their personal information, participants were requested for verbal consent; for those patients unable to speak, consent was requested from their relatives. For those who could write and read, written consent was taken.

### Operational definition of terms

**Active smokers:** refers to patients smoking a cigarette in the past 3 months before the current hospital admission [31].

**Adherence (inhalation techniques):** is the degree to which known asthma and COPD patients’ medication-taking behavior (inhalation techniques steps) corresponds with agreed recommendations from a healthcare provider. Good adherence was defined as an average adherence to study medications of >80% over the whole period the subject was in the study except critical steps. Poor adherence (not adhere to inhalation techniques) was defined as average adherence to inhaled medication ≤80% including critical steps or missed at least one critical steps [32].

**Critical step**: procedure if it is missed which leads to poor asthma control. These are place mouthpiece between teeth and lips, simultaneously press canister and breathing in slowly and inhaler out of mouth and hold breathe for 5–10 sec [33].

**Efficient:** Refers when patients can perform all critical steps regardless of non-critical steps [34].

**Inefficient:** Refers when patients miss any one or more critical steps regardless of performance of non-criticalsteps [34].

**Index admission:** The first occurrence of the patient’s hospital encounter for asthma or

COPD within the study period [35] and inpatient stay discharged alive with no missing length of stay (LOS) [36].

**Readmission** is an inpatient stay that begins within 30 days of the discharge date of an index admission.

**The readmission rate** is the percentage of index admissions that are readmitted within 30 days [36].

**30-day readmission timing:** any unplanned consecutive admission within 30 days (1–30) after being discharged alive from a hospital stay where asthma or COPD was a principal diagnosis during the study period [35].

**Spirometer**: the apparatus commonly used to measure the volume of air exchanged during breathing and the respiratory rate.

**Forced vital capacity (FVC):** the maximum volume of air in the lung after a forceful exhalation following forceful inhalation.

**Forced expiratory volume (FEV**_**1**_**)-**the volume of air expired forcefully in one second.

**Peak expiratory flow rate (PEFR)-**a maximum expiratory air flow beyond which the flow cannot be increased any more even with greatly increased additional force.

**FEV**_**1**_**/FVC (FEV**_**1**_**%):** the percentage of the FVC expired in the first second of maximal forced expiration following full inspiration. Predicted values greater than 80% is usually considering as normal.

**Obstructive disorder:** FEV_1_/FVC ratio <0.7 and percent predicted FEV_1_<80% predicted normal, FVC value reduced or normal.

**Chronic obstructive respiratory diseases:** are obstructive lung diseases that include either asthma or COPD (ICD-10, 2019).

**Long-term oxygen therapy:** use of oxygen for greater or equal to 16hrs per days and for multiple days (GOLD-2019).

## Results

### Characteristics of study cohort

**Fig 1:**
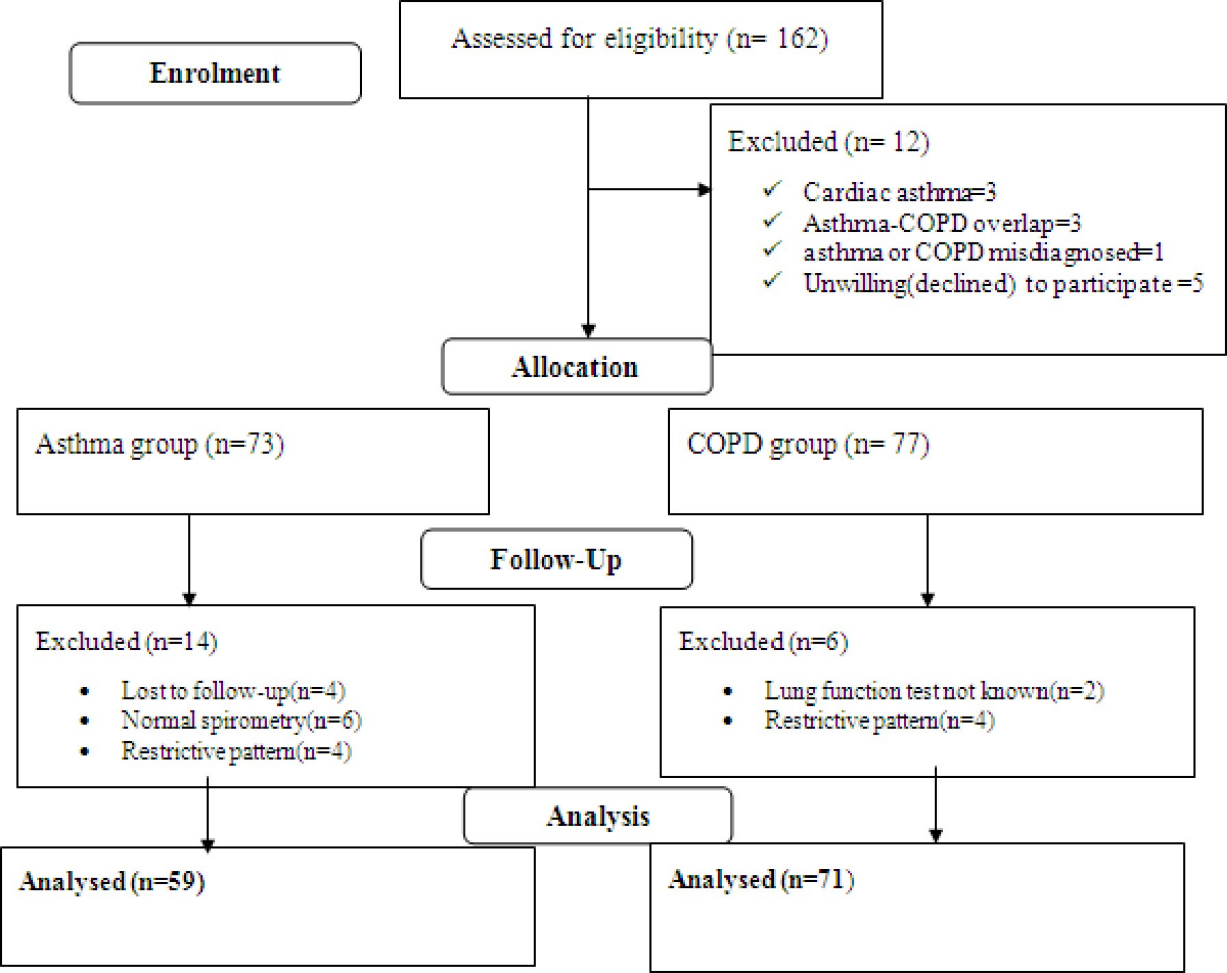
Flow diagram of study participants (asthma/COPD patients) at JMC from April-September, 2019.

In total, 162 asthma/COPD adult patients were admitted to hospital for acute exacerbations. Twelve patients were excluded from enrolment because of not meeting inclusion criteria and not willing to participate. During the follow-up period, 20 patients were excluded due to loss to follow-up, 4 patients, 14 patients not obstructive, and two patients lung function test unknown. One hundred thirty patients met the eligibility criteria (asthma = 59, COPD = 71) and included into the final analysis. Eligible participants were followed for a median period of 5 days (IQR, 3–14 days). (Fig 1).

### Description of socio-demographic characteristics

Of 130 eligible patients with chronic obstructive respiratory disease, 78(60%) were male. The median age of patients was 59(IQR, 50–70) years. Sixty-eight (52.31%) patients were from rural areas. Majority, 73(56.15%) of patients had no formal education (could not read and write). Seventy 70 (53.85%) patients had history of living with pet animals (cats, dogs). Twenty-eight (21.54%) were active smokers. Of these, 25(35.21%) were COPD patients, and 3(5.08%) were asthmatic. Twenty three (82.14%) of the smokers consume1-10 cigarettes per day. At baseline, 49 (37.69%) of patients had low body mass index (BMI<18.5kg/m^2)^. (Table 1).

**Table 1:** Baseline socio-demographic characteristics of study cohort at JMC, April-September, 2019.

Variables		Asthma (N = 59) n (%)	COPD (N = 71) n (%)	Overall(N = 130) n (%)	χ^2^p-value
Gender	Male	29 (49.15)	49 (69.01)	78(60.00)	0.021
Age	Median(in years)	53(IQR,35–61)	60(IQR,53–70)	59(IQR,50–70	0.145
BMI	<18.5kg/m^2^	14 (23.73)	35(49.30)	49(37.69)	0.003
	≥18.5kg/m^2^	45(76.27)	36(50.70)	81(62.31)	
Marital status	Single	5 (8.47)	3(4.23)	8(6.15)	0.604
	Married	50 (84.75)	63(88.73)	113(86.92)	
	Widowed	4 (6.78)	5(7.04)	9(6.92)	
Residence	Urban	32 (54.24)	30(42.25)	62(47.69)	0.173
	Rural	27(45.76)	41(57.75)	68(52.31)	
Educational level	Can’t read & write	26(44.07)	47(66.20)	73(56.15)	0.040
	Primary	17(28.81)	13(18.31)	27(20.77)	
	Post-primary	16(27.12)	11(15.49)	30(23.08)	
Occupation	Employed	25(42.37)	26(36.62)	51(39.23)	0.658
	Farmer	18(30.51)	27(38.03)	45(34.62)	
	Housewife	16(27.12)	18(25.35)	34(26.15)	
Living status	With family	56 (94.92)	67(94.37)	123(94.62)	0.890
	Alone	3(5.08)	4(5.63)	7(5.38)	
Live with pet animals	Yes	35(59.32)	35(49.30)	70(53.85)	0.254
Smoking status	Active smokers	3(5.08)	25(35.21)	28(21.54)	<0.001
	Non-smokers	46(77.97)	32(45.07)	78 (60.00)	
	Ex-smokers	10(16.95)	14(19.72)	24(18.46)	
Active smokers cig/day	1–10	3(100.00)	20 (80.00)	23(82.14)	0.393
	11–20	0(0)	5(20.00)	5(17.86)	

*BMI = Body mass index, IQR = inter quartile range

### Clinical characteristics

Of total patients,111(85.38%) had history of previous chronic obstructive respiratory disease with the median duration of 3 years (IQR, 2–6 years) for COPD, and 10 years (IQR, 4.5–19 years) for asthma. The overall median length of hospital stay was 5 days (IQR, 3–14 days). The median length of hospital stay for acute exacerbation of asthma was 3 days (IQR, 3–6 days); while for acute exacerbation of COPD was 8 days (IQR, 4–19 days). Seventy-nine (60.77%) patients had history of hospital admission and nearly half, 38(48.10%) of them had two admissions per annum. Eight (6.15%) patients had history of ICU admission (asthma = 4, COPD = 4).

Fifty-one (39.23%) patients had history of oxygen therapy prior to the current study, of which 15(29.41%) were on oxygen for ≥16hrs. Majority, 117(90%) of patients had history of difficulty in falling asleep during night, to which asthma accounted for 57(48.72%) patients and 60 (51.28%) were accounted for COPD patients. According to GINA guideline, 34(29.06%) patients encountered severe night attacks, 7*/week. Sixty (46.15%) patients had history of limitation to perform daily activities (asthma = 26, COPD = 34) (Table 2).

**Table 2:** Baseline clinical characteristics of study cohort at JMC, April-September, 2019.

Variables		Overall(N = 130) N (%)	Asthma (N = 59) N (%)	COPD(N = 71) N (%)	p -value
Duration of chronic respiratory disease(year)	Known	111(85.38)	10 (IQR,4.5–19)	3 (IQR,2–6)	0.756
	New	19(14.62)	Newly admitted	Newly-admitted	
History of hospital admission	Yes	79(60.77)	34(57.63)	45(63.38)	0.504
Number of hospital admission per year	1*/year	24(30.38)	9(26.47)	15(33.33)	0.017
	2*/year	38(48.10)	22(64.71)	16(35.56)	
	≥ 3*/year	17(21.52)	3(8.82)	14(31.11)	
History of ICU admission	Yes	8(6.15)	4(6.78)	4(5.63)	0.787
History of oxygen therapy(prior to the current study)	Yes	51(39.23)	23(38.98)	28(39.44)	0.958
duration of oxygen therapy /day	<16hrs	36(70.59)	15(65.22)	21(75.00)	0.046
	≥16hrs	15(29.41)	8 (34.78)	7(25.00)	
Difficulty in falling asleep	Yes	117(90.00)	57(96.61)	60(84.51)	0.022
Frequency of night attacks	3–4*/month	83(70.94)	39(68.42)	44(73.33)	0.164
	7*/week	34(29.06)	18(31.58)	16(26.67)	
Limit performing daily activities	Yes	60(46.15)	26(44.07)	34(47.89)	0.133
Last hospitalizations	≥12 month	34(26.15)	15(25.42)	19(26.76)	0.970
	<12 month	45(34.62)	19(32.20)	26(36.62)	
	Not-admitted	51(39.23)	25(42.37)	26(36.62)	
Chest clinic physician visit	≥12 month	10(7.69)	4(6.78)	6(8.45)	0.229
	<12 month	77(59.23)	34(57.63)	43(60.56)	
	Never visit	43(33.08)	21(35.59)	22(30.99)	

#### ICU-intensive care unit

**Fig 2:**
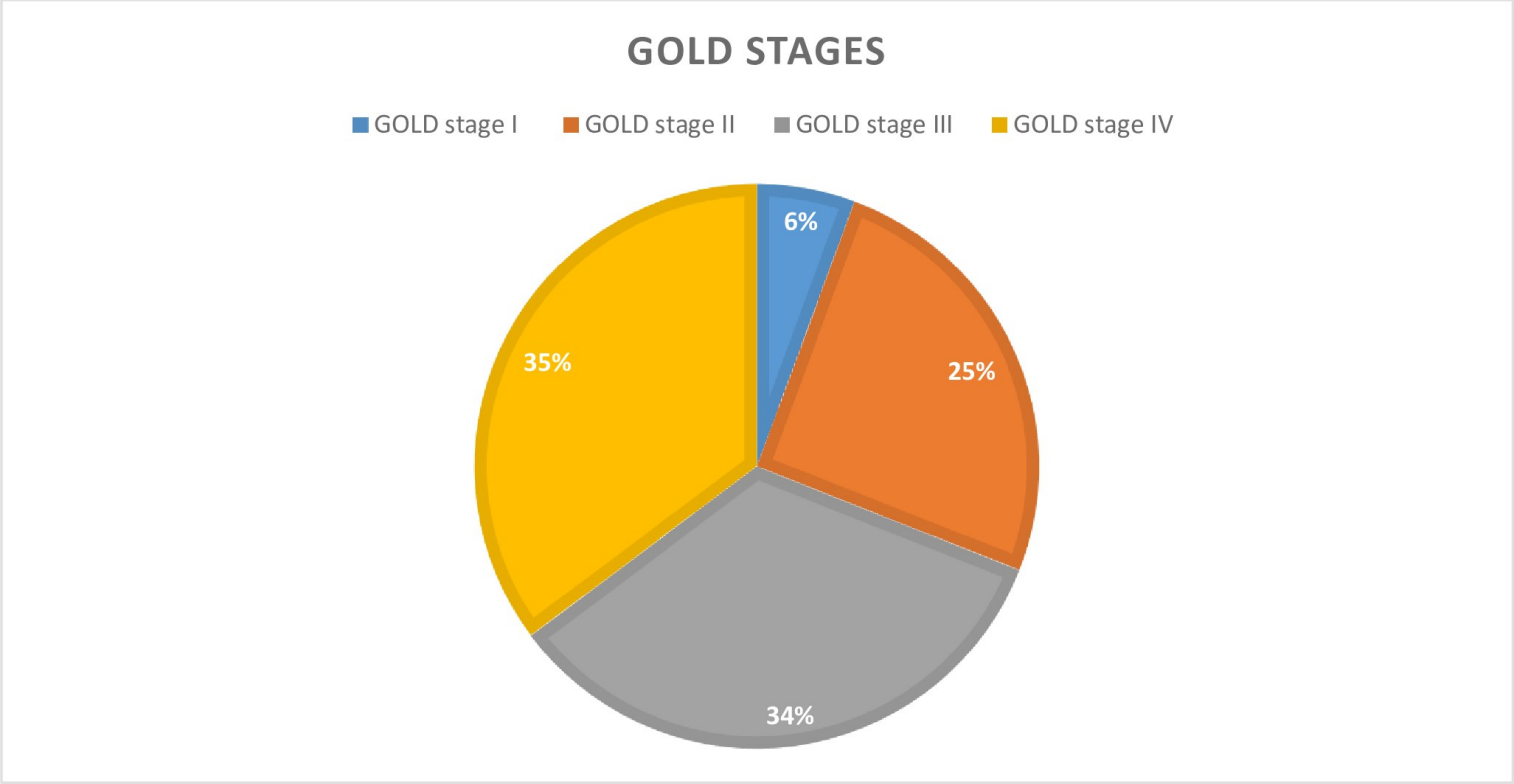
Clinical staging for COPD using GOLD at JMC, April -September, 2019.

Based on GOLD severity staging, 71COPD patients, 25(35.21%) were GOLD stage IV and 24(33.80%) were GOLD stage III (Fig 2).

**Fig 3:**
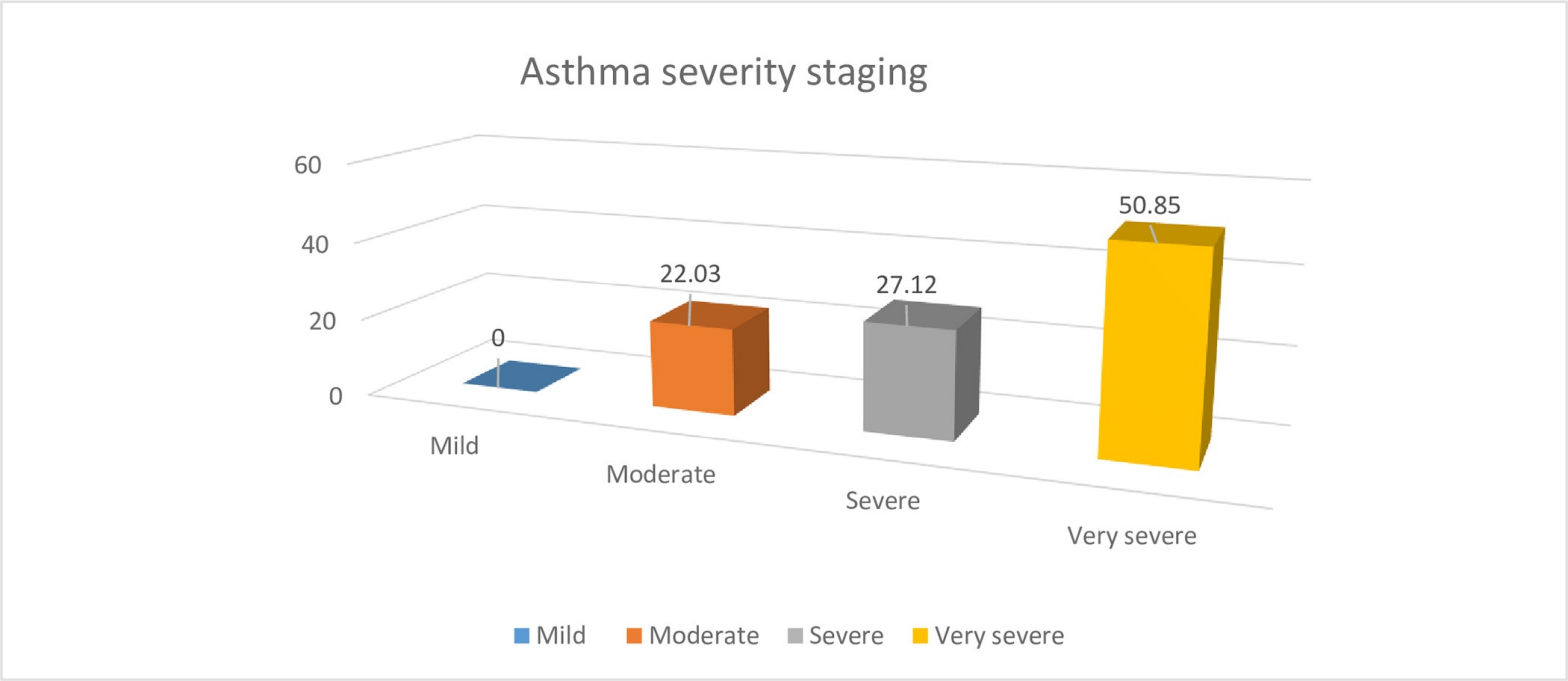
Clinical staging for asthma using GINA guideline at JMC, April -September, 2019.

According to GINA staging severity, 30(50.85%) had severe asthma and 16(27.12%) had very severe asthma (Fig 3).

Sixty-one (46.92%) patients (asthma = 20, COPD = 41) had co-morbidities. Of these, pulmonary hypertension, 34(47.89%), severe community-acquired pneumonia (SCAP), 30(42.25%), and heart failure (IHD), 23(32.39%) were the most common in COPD patients, while SCAP 32(54.24%), hypertension, 6(10.17%), and heart failure, 6(10.17%) were mostly seen in asthma patients (Table 3).

**Table 3:** Comorbid conditions among study cohort at JMC, April-September, 2019.

Variables		Overall(N = 130)	Asthma (N = 59)	COPD(N = 71)	p -value
		N (%)	N (%)	N (%)	
Common co-morbidity (n = 61)	yes	61(46.92)	20(33.90)	41(57.75)	0.007
Heart failure(IHD)	yes	29(22.31)	6(10.17)	23(32.39)	0.002
Hypertensions	yes	17(13.08)	6(10.17)	11(15.49)	0.370
Diabetes mellitus	yes	4(3.08)	1(1.69)	3(4.23)	0.406
Cardiac arrhythmia	yes	7(5.38)	1(1.69)	6 (8.45)	0.089
Depression	yes	1(0.77)	0(0)	1(1.41)	0.360
Pulmonary HTN	yes	39(30.00)	5(8.47)	34(47.89)	0.000
Renal failure	yes	8(6.15)	0(0)	8(11.27)	0.008
Cancer	yes	4(3.08)	2(3.39)	2(2.82)	0.851
DVT	yes	2(1.54)	1(1.69)	1(1.41)	0.895
Pneumonia(SCAP)	yes	62(47.69)	32(54.24)	30(42.25)	0.173

SCAP: severe community acquired pneumonia, IHD: ischemic heart disease, DVT: deep vein thrombosis, HTN: hypertension

### Drug-related and investigation findings

Of the total admitted patients, 100 (76.92%) patients had been using salbutamol inhaler since diagnosis. Eighty-three (63.85%) patients had history of antibiotics usage and 51 (39.23%) patients had history of long-term oxygen therapy≥ 16hrs per day (asthma = 22, COPD = 29). The most frequently used drugs during hospital stay were salbutamol inhaler, 128(98.46%), oxygen therapy, 94(72.31%), and prednisolone, 81(62.31%). The most frequently used antibiotics were ceftriaxone injection, 97(74.62%) and azithromycin, 91(70%).

With regard to laboratory findings, twenty (15.38%) patients had elevated haemoglobin (asthma = 2, COPD = 18). Fifty-nine (45.38%) patients presented with raised white blood cells. Creatinine was also increased in 21(16.5%) patients and blood urea nitrogen also elevated in 16 (12.31%) patients (asthma = 5, COPD = 11) (Table 4).

**Table 4:** Drug-related and laboratory variables recorded at JMC, April-September, 2019.

Variables		Over all (n, (%)		Asthma (n, (%)	COPD (n, %)	P-value
Past Antibiotics uses history		yes	83(63.85)	33(55.93)	50(70.42)	0.087
Past Long term O2 therapy(≥16hrs)		yes	51(39.23)	22(37.29)	29 (40.85)	0.679
Past Salbutamol inhaleruse history		yes	100(76.92)	48(81.36)	52 (73.24)	0.274
Past selmaterol inhaler		No	129(99.23)	58(98.31)	71(100.00)	0.271
Past Bechlomethasone inhaler		yes	33(25.38)	13(22.03)	20(28.17)	0.424
Prednisolone tablet		yes	35(26.92)	14(23.73)	21(29.58)	0.454
Baseline oxygen saturations		<90%	83(63.85)	32(54.24)	51(72.86)	0.038
		≥90%	47(36.15)	27(45.76)	20(28.17)	
Current medications(on admission)						
Oxygen therapy		yes	94(72.31)	40(67.80)	54(76.06)	0.295
Duration of oxygen therapy		<16hrs	77(81.91)	35(87.50)	42(77.78)	0.226
		≥16hrs	17(18.09)	5(12.50)	12(22.22)	
Salbutamol inhaler		yes	128(98.46)	58(98.31)	70 (98.59)	0.895
Salmeterol inhaler		yes	5(3.85)	2(3.39)	3 (4.23)	0.805
Bechlomethasone inhaler(ICS)		yes	57(43.85)	22(37.29)	35(49.30)	0.170
Prednisolone tablet		yes	81(62.31)	35(59.32)	46(64.79)	0.522
Hydrocortisone injections		yes	43(33.08)	21(35.59)	22(30.99)	0.286
Budesonide+formoterol inhalations		No	125(96.15)	59(100.00)	66(92.96)	0.038
Drugs for comorbidities						
Ceftriaxone		yes	97(74.62)	42(71.19)	55(77.46)	0.413
Azithromycin		yes	91(70.00)	40(67.80)	51(71.83)	0.617
Vancomycin		yes	5(3.85)	1(1.69)	4(5.63)	0.245
Doxycycline		yes	3(2.31)	0(0)	3(4.23)	0.110
Laboratory parameter						
Hgb g/dl	High	20(15.38)		2(3.39)	18(25.35)	0.001
	Normal	103(79.23)		56(94.92)	47(66.20)	
	Low	7(5.38)		1(1.69)	6(8.45)	
WBC	High	59(45.38)		28(47.46)	31(43.66)	0.851
	normal	68(52.31)		30(50.85)	38(53.52)	
	low	3(2.31)		1(1.69)	2 (2.82)	
Serum creatinine	High	21(16.15)		6(10.17)	15(21.13)	0.237
	normal	92(70.77)		45(76.27)	47(66.20)	
	low	17(13.08)		8(13.56)	9(12.68)	
BUN	High	16(12.31)		5(8.47)	11(15.49)	0.079
	normal	55(42.31)		21(35.59)	34(47.89)	
	Not measured	59(45.38		33(55.93)	26 (36.62)	

Hgb-hemoglobin, WBC- white blood cell, BUN-blood urea nitrogen

#### Spirometer results for pre and post bronchodilator

On admission, lung function test was performed for all candidate patients. The FEV_1_/FVC was <0.7 in 130 patients showing obstructive pattern. The relationship between pre-bronchodilator values and bronchodilator response was estimated and declared as acute exacerbation of asthma or COPD (severity).

### Measurements of inhalation techniques adherence

**Fig 4:**
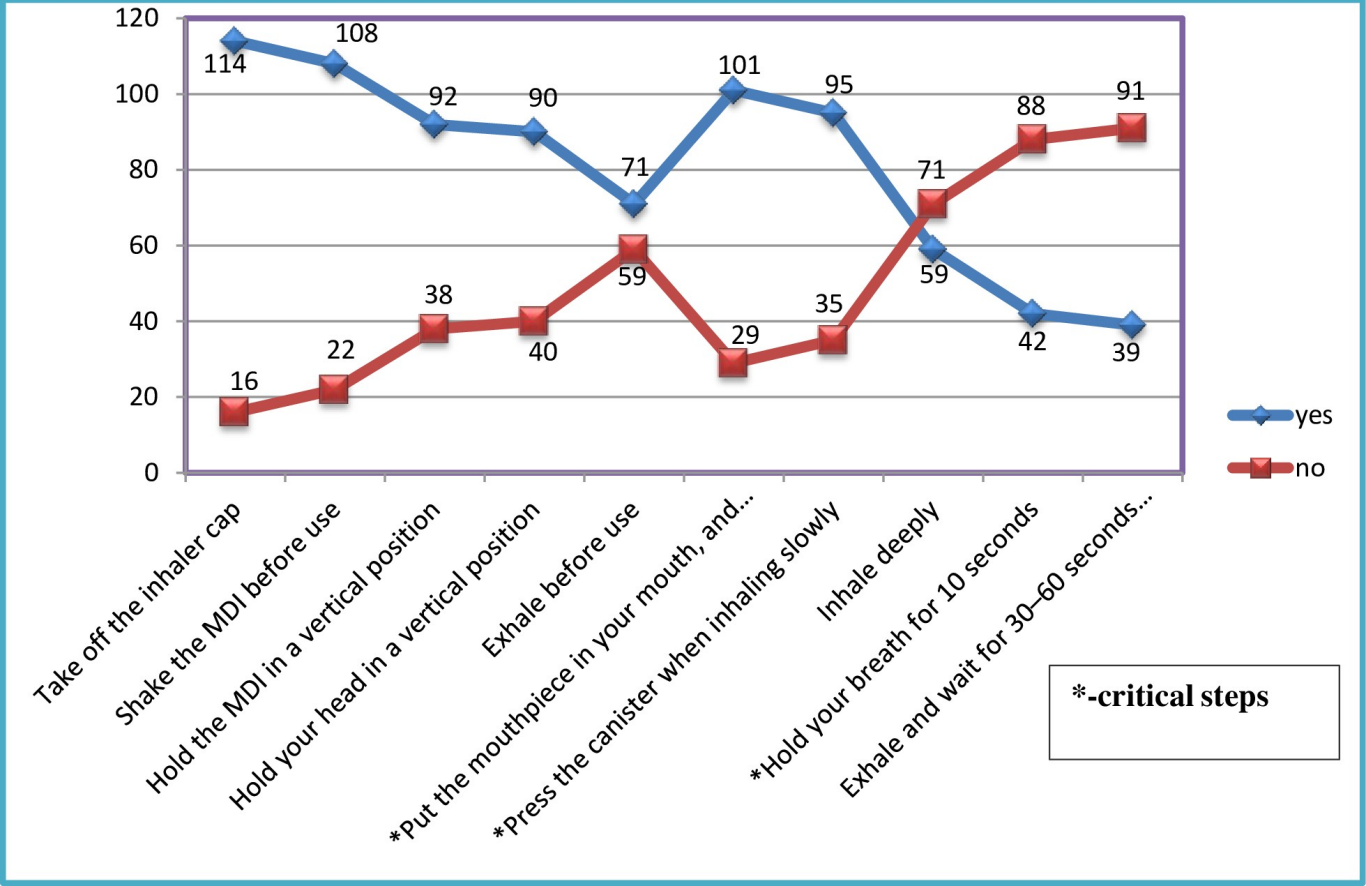
Instructions measurements for MDI (step-wise correct use of the inhalation techniques) at JMC, April-September, 2019. *MDI-metered dose inhalations.

For all eligible patients, 10 step-wise inhalation techniques were provided to check the adherence of effective inhalations. Fourteen (10.78%) patients totally could not perform inhalation techniques correctly including the three critical steps and declared as “inefficient”. The remaining 116(89.23%) patients missed at least one steps, except the three critical inhalation techniques and declared as “efficient”. As depicted in Fig 4, 114 patients performed step one correctly (blue color), 16 patients cannot perform correctly (red color), and so on. As steps goes up, the ability to remember and perform effectively was decreased i.e. 91(70%) could not perform correctly the last steps (exhale and wait for 30–60 seconds before the other inhaler) (red color). (Fig 4).

### Rate of 30-days readmission

Of 130 admitted patients, 21(18.10%) had a new AE of asthma and COPD that required hospitalization in the 30-days after discharge. Fourteen (12.07%) of them were asthmatic and 7(6.03%) were COPD patients. The total analysis time at risk was 3,705 days, making an overall incidence rate of 30-day readmission 55.8 per100, 000 person-years, (95% CI, [36.4–85.6]). The incidence rate of 30-day readmission for acute exacerbation of COPD was 59.9 per 100,000 person-years, 95% CI [35.9–158.3), whereas that of asthma was 189.9 per100, 000 person-years, 95% CI [112.5–320.8].

**Fig 5:**
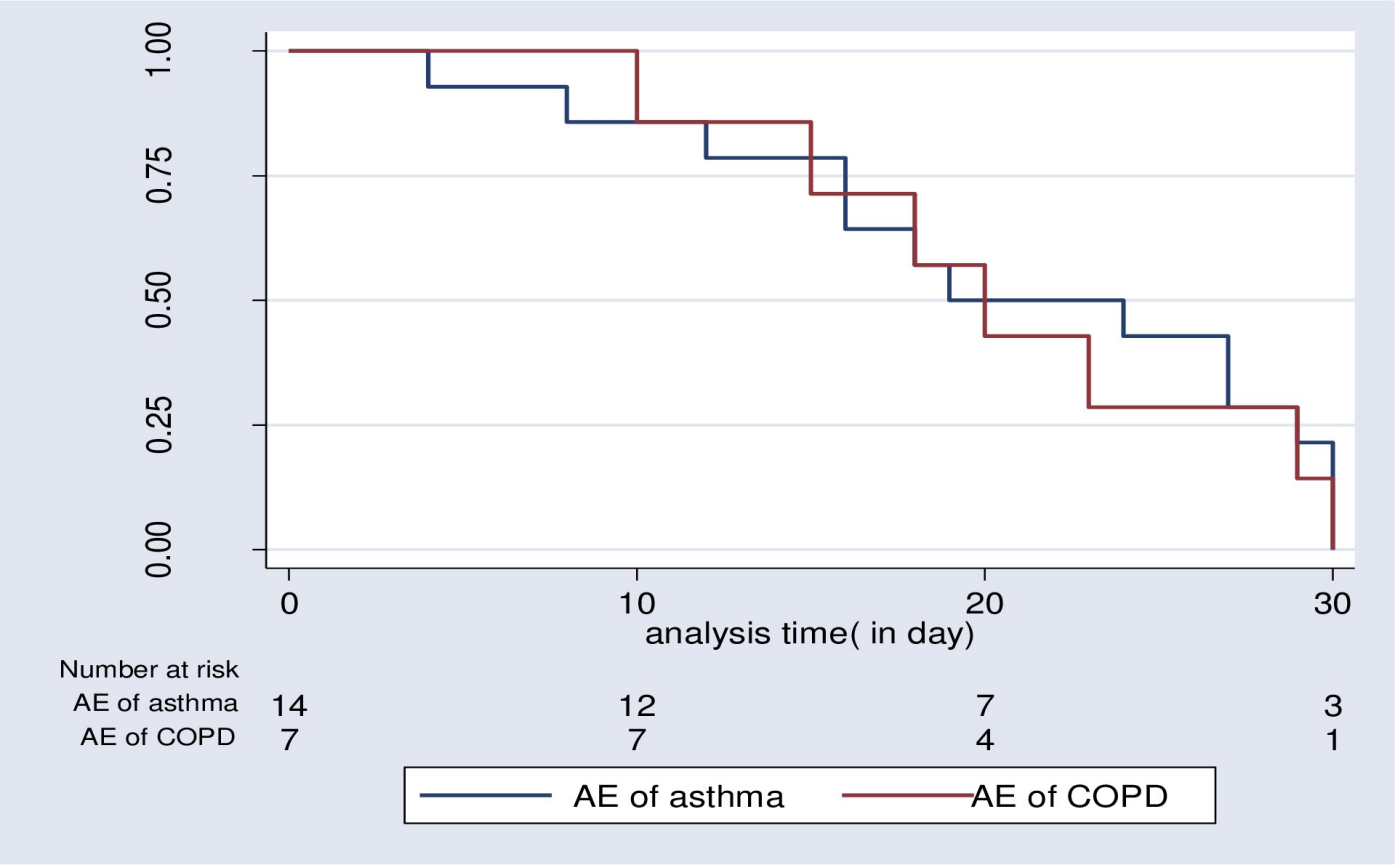
Survival estimates for 30-day readmission among patients admitted with acute exacerbations of COPD or asthma at JMC, April-September, 2019.

The overall median time to 30-day readmission was 20 days (IQR, 16–29). The median time to 30-day readmission for AECOPD was 20 days (IQR, 15–29), while 21.5days (IQR.16-29) for acute exacerbation of asthma, (p = 0.773). (Fig 5).

### Predictors of 30-day readmission

Several variables such as history of hospital admission, use of high dose salbutamol inhaler, chest clinic physician visit, occupational status, residence, and other four variables (age, BMI, smoking status, and educational status) were omitted from analysis due to violation of cell adequacy test and two variables (comorbidity and living status) removed due to co-linearity effect. Therefore, nine variables were candidates for bivariate Cox-regression analysis (p<0.25) depicted in Table 5. Further multivariate Cox-regression identified prolonged use of oxygen therapy and frequent hospitalization per yearto be independent predictors of 30-day readmission.

**Table 5:** Crude and adjusted Cox-proportional hazard regression for predictors of 30-days readmission of the cohort at JMC, April- September, 2019.

Variables	30-day readmission		CHR[CI 95%]	p-value	AHR[CI 95%]	P-value
	category					
Bechlomethasone inhalation	No		1		1	
	yes		2.42[1.021–5.756]	0.045	2.426[0 .680–8.651]	0.172
Number of hospitalization/year	1*/year		1		1	
	2*/year		7.69[0.99–59.57]	0.051	6.467[0.768–54.446]	0.086
	≥3*/year		11.86[1.458–96.53]	0.021	11.482[1.308–100.793]	0.028*
antibiotics	No		1		1	
	yes		2.529[0.851–7.516]	0.095	0.418[0.093–1.879]	0.256
LTOT	<16hrs		1		1	
	≥16hrs		5.818[2.129–15.892]	0.001	4.972 [1.041–23.736]	0.044*
daily activities	No		1		1	
	yes		3.175[0.703–14.336]	0.133	1.140[0.377–3.444]	0.816
Frequency of Night attacks	3–4*/mnth		1		1	
	7*/week		0.235[0.031–1.808]	0.164	1.151[0.338–3.921]	0.822
Baseline O2 saturation	≥90%		1		1	
	<90%		1.897[0.695–5.179]	0.211	0.780[0.176–3.458]	0.744
Prednisolone tablet	No		1		1	
	yes		1.781[0.738–4.298]	0.199	0.623[0.193–2.011]	0.274
FEV_1_			0.987 [0.962–1.012]	0.131	1.0156[0.988–1.044]	0.274

LTOT = long term oxygen therapy, Mnth-month, FEV_1_: Forced expiratory volume in 1 sec, AHR—Adjusted Hazard Ratio, COR-Crude Odd Ratio, CI- Confidence interval

Accordingly, patients who were on oxygen therapy for ≥16hrs per 24hrs had 5 times increased hazard of 30-day readmission (AHR = 4.972, 95% CI [1.041–23.736]. Similarly, patients with three times or greater admissions per year had 11 times increased hazard of 30-day readmission (AHR = 11.482[1.308–100.793]) (Table 5).

## Discussion

This prospective cohort study summarized the rate of 30-day readmission and it’s predictors among patient treated for acute exacerbation of chronic obstructive respiratory disease, asthma, and COPD. During the study period, 21(18.01%) patients were readmitted, making an overall incidence rate of 55.8 per100, 000 person-years (COPD = 7(6.03%), asthma = 14(12.07%). It was 59.9 per 100,000 person-year, 95% CI, [35.9–158.3), and 189.9 per100, 000 person-year, 95% CI [112.5–320.8] for AE of COPD and Asthma, respectively. The overall median survival time to 30-day readmission was 20 days (IQR, 16–29). The median survival time to 30-day readmission for AECOPD was 20 days (IQR, 15–29), and that of AE of asthma was 21.5 days (IQR.16-29). Prolonged use of oxygen therapy and frequent hospital admissions were independent predictors for 30-day readmission.

Regarding an overall rate of 30-days readmission, our finding (21(18.01%)) was almost similar with studies conducted in Chicago [37], Ohio [38], Spain [26], and USA [39] which revealed an overall 30-days readmission rate of 18.5%, 18.1%, 17.98%, and 19.4% respectively. However, it’s inconsistent with studies conducted by Buyantseva et al [40], and Meservey et al [41] that reported a higher 30-days readmission rate of 39%, and 23% respectively. Similarly, a lower 30-days readmission rate was reported in USA (9.2%) and UK (4.7%) [42]. This variation might be due to difference in socio-economic status, sample size, and duration of study period (6 months vs. greater than one year).

In this study, one hundred sixteen (89.23%) patients were adherent (efficient) to inhalation technique. This adherence rate is relatively better than the study conducted by Kebede et al, 87(62.14%) [28]. In a study conducted in Turkey, nearly 23% of the patients had suboptimal adherence to inhalation technique [43]. Difference in the methodology used to measure adherence level might contribute to the variation. Furthermore, the direct involvement of clinical pharmacists in the patient care process and regular provision of counseling on medication use in our set up may contributed for better adherence.

Although we have evaluated a wide range of risk factors for hospital readmission, only two clinical factors were found to be independent predictors of 30-days readmission in the current study. In our study, patients who were on oxygen therapy for ≥16hrs per day had 5 times increased hazard of 30-day readmission (AHR = 4.972.95% CI [1.041–23.736]. A number of previous similar studies [39, 41], have also reported long term oxygen therapy as a risk factor associated with an increased risk of readmission among patients with chronic obstructive airway diseases. Individuals who stay longer on oxygen therapy likely have severe and advanced disease and are at higher risk for hospital readmission. Furthermore, patients who admitted to the hospital three times or greater in the previous year had 11 times increased hazard of 30-day readmission (AHR = 9.5, 95% CI [1.12–80.16]. This finding is in agreement with several other studies [40, 42, 44, 45]. According to Guerrero M et al., [45], the rate of 30 days readmission was significantly higher in COPD patients with a history of >2 exacerbations in the previous year. Frequent hospital admission is an indicator of disease severity and patients with advanced disease are very prone for readmission.

No association was found between FEV_1_% predicted and the risk of asthma and COPD readmission. This finding supports the result of a previous study by Bahadori et al., [29] that found no differences between FEV_1_% predicted and the risk of COPD readmission. However, other studies [27, 30, 46] found FEV_1_<50% predicted to be predictive of a higher risk of 30-days readmission. The possible justification for the lack of association in the current study might be related to the small sample size, which reduces statistical power to find true association.

The prevalence of COPD/asthma in Ethiopia is comparable with neighboring Sub-Saharan African countries. A prevalence rate of 17.6% was reported for COPD [47] and this is in-line with studies done in Uganda [48] and Tanzania [49]. The prevalence of asthma was reported to be 29.6% [50]. This prevalence was relatively higher compared to the study conducted in Egypt [51], and Uganda [52]. Differences in climate conditions, growing population size, and radical urbanization might contribute to the increment in the prevalence of asthma.

### Limitations

The findings of the present study should be interpreted in the context of several possible limitations. First, inclusion of a small number of patients might under power the study. Second, follow-up period of 6 months may be relatively short, but we focused on early 30-days readmission. Third, merging two populations of obstructive diseases may affect the validity of the outcome. Moreover, salbutamol alone was used to test the lung functions in the study due to unavailability of ipratropium, as most of bronchodilator reversibility studies were conducted using the combinations of short-acting beta agonists and anti-muscarinic. Finally, the present study was conducted in a single institution, and so the extrapolation of these findings to other settings must be done with care.

## Conclusion

In conclusion, hospital readmission rates for AE of asthma and COPD within 30 days of discharge were alarmingly high. Frequent hospital admission and long-term oxygen therapy during hospital stay were independent predictors of 30-day readmission.

## Supporting information

S1 File (DOCX)

S2 File (DOCX)

## Abbreviations

AE: Acute Exacerbations
COPD: Chronic Obstructive Pulmonary Disease
AHR: Adjusted Hazard Ratio
AOR: Adjusted odd ratio
FEV_1_: Forced Expiratory Volume in One Second
IQR: Inter quartile range
SABA: Short Acting Beta Agonist
LTOT: Long-term oxygen therapy
JMC: Jimma Medical Center
