# Supplementary material for: Early readmission and its predictors among patients treated for acute exacerbations of chronic obstructive respiratory disease in Ethiopia: A prospective cohort study
Source: PLoS One. 2020 Oct 6;15(10):e0239665. doi: 10.1371/journal.pone.0239665 (PMC7537865; doi:10.1371/journal.pone.0239665)
Supplement: S1 File — (DOCX) [file pone.0239665.s001.docx]

JIMMA UNIVERSITY

INSTITUTE OF HEALTH

SCHOOL OF PHARMACY

1. **Participant written informed consent form**

Dear research participant,

I am **Zenebe Kano** from school of pharmacy and Masters Student in clinical pharmacy. I am conducting a research on admission outcomes and rates of 30-day readmission of asthma or COPD. The aim of this study is to assess admission outcomes, rate of 30-day readmission, and outcome predictors among patients hospitalized with acute exacerbations of asthma and COPD”at JUMC. The finding generated by this study would help health care professionals to stratify patients for appropriate management that could optimize patient outcomes and reduce re-hospitalizations.

Your participation in the study is voluntary and that you can choose not to be included in the study or withdraw at any time. Your refusal not to participate will in no way affect your service at the hospital. All personal identifiers will be removed and no personal information will be forwarded to others. You may not personally derive any benefits directly from participating in the study and also there is no any risk or harm that this research will bring to you.

Your personal information was maintained through use of unique codes and restricted access to the data set to the principal investigator and those working with him. I am very much grateful for your keen interest and honesty in sharing information. Whenever you have any questions or comments please contact Zenebe kano: phone No: 0912812388. Email: [zenebekano21@gmail.com](mailto:zenebekano21@gmail.com)

Date:________ Signature of interviewer ___________ Signature of respondent _____________

## Data collection format

- **Data collection tool to assess** treatment outcomes, causes and rates for 30-day readmission in patients hospitalized with acute exacerbations of Asthma and COPD

***Instruction***

A. Select your answer for the questions by encircling the choice provided

B. If your answer is out of the choice provided; write it in the space provided

- **Section I. Socio-demographic Characteristics**

This section asks your personal information. Please answer every question accordingly.

| No. | | Question | | | | Response | | | **Units**=________________________ | | | | | | Remark | | |  |  |
| --- | --- | --- | --- | --- | --- | --- | --- | --- | --- | --- | --- | --- | --- | --- | --- | --- | --- | --- | --- |
|  | | Gender | | | | 1. Male 2. Female 4.card number______________  **3. phone No=**__________________________  5. Date of admission---------------------------------------- | | | | | | | | |  | | |  |  |
|  | | Age | | | | 1.______years 2. Weight(kg)______  3. Height______ 4. BMI_________ | | | | | | | | |  | | |  |  |
|  | | Marital status | | | | 1. Single 2. Married 3. Widow 4. Divorced | | | | | | | | |  | | |  |  |
|  | | Residence | | | | 1. Urban 2. Rural | | | | | | | | |  | | |  |  |
|  | | Education status | | | | 1. Can’t read and write 2. Can read and write but no formal education 3. Primary 4. Secondary 5. college and higher | | | | | | | | |  | | |  |  |
|  | | Occupation | | | | 1. Governmental employee 2.students 3.Merchant 4.Farmer 5.Housewife 6. Daily laborer 7.industrial workers 8. Other ……………… | | | | | | | | |  | | |  |  |
|  | | Home status | | | | 1. With Family 2. alone | | | | | | | | |  | | |  |  |
|  | | live with Pet animals | | | | 1. Yes 2. No | | | | | | | | |  | | |  |  |
|  | | smoking status | | | | 1. Smokers 2. Non-smokers 3. Ex-smokers | | | | | | | | |  | | |  |  |
|  | | 1f smokers for Q10 | | | | 1.______packs/day(estimate)  2. ______packs/month(estimate)  3. ______.packs/year(estimate) | | | | | | | | |  | | |  |  |
| **Section II. Medical History (for only cases)** | | | | | | | | | | | | | | | | | |  |  |
|  | | Duration of chronic respiratory disease | | | | 1. COPD_______month/year  2. Asthma________ month/year  3. ACOS_________________ month/year  4.newly admitted-----------------days | | | | | | | | |  | | |  |  |
|  | | History of hospital admission | | | | 1. Yes 2. No  If yes, date of admission------------------------------- | | | | | | | | |  | | |  |  |
|  | | If yes to Q13, | | | | - reason for previous admission:   1. For asthma 2. For COPD 3. For ACOS 4.For other specify____________ | | | | | | | | |  | | |  |  |
|  | | If yes for Q.13, for how long stayed | | | | ­­­­­­­­­­­­­­­­­­_________Days or___________month/years | | | | | | | | |  | | |  |  |
|  | | Previous history of ICU admission. | | | | 1. Yes 2.No | | | | | | | | |  | | |  |  |
|  | | History of Oxygen therapy | | | | 1. Yes 2.No | | | | | | | | |  | | |  |  |
|  | | If yes to Q,17 | | | | Duration on oxygen per day_______(estimate in hrs/24hours | | | | | | | | |  | | |  |  |
|  | | Difficulty in falling asleep | | | | 1. Yes 2.No | | | | | | | | |  | | |  |  |
|  | | If yes to Q.19 | | | | Frequency of Night attacks_______ ( mention) | | | | | | | | |  | | |  |  |
|  | | Need for assistance for performing daily activities | | | | 1. Yes 2.No | | | | | | | | |  | | |  |  |
| **Section III: clinical characteristics and related factors**  **1. Clinical presentations(sign and symptoms)**  **a. Cough** | | | | | | | | | | | | | | | | | |  |  |
|  | | Do you usually have a cough? (Count a cough with first smoke or on first going out-of-doors) | | | | | | | | | | 1. Yes 2.No | | | |  | | |  |
|  | | Do you usually cough as much as 4 to 6 times a day, 4 or more days out of the week? | | | | | | | | | | 1. Yes 2.No | | | |  | | |  |
|  | | Do you usually cough at all on getting up, or in the morning? | | | | | | | | | | 1. Yes 2.No | | | |  | | |  |
|  | | Do you usually cough at all during the rest of the day or at night? | | | | | | | | | | 1. Yes 2.No | | | |  | | |  |
|  | | Do you usually cough on most days for 5 consecutive months or more | | | | | | | | | | 1. Yes 2.No | | | |  | | |  |
| **b. Phlegm** | | | | | | | | | | | | | | | |  |  |  |  |
|  | | Do you usually bring up phlegm from your chest? (Count phlegm with the first smoke or on first going out-of-doors | | | | | | | | | | | 1. Yes 2.No | | |  | | |  |
|  | | Do you usually bring up phlegm like this as much as twice a day, 4 or more days out of the week? | | | | | | | | | | | 1. Yes 2.No | | |  | | |  |
|  | | Do you usually bring up phlegm at all on getting up or first thing in the morning? | | | | | | | | | | | 1. Yes 2.No | | |  | | |  |
|  | | Do you usually bring up phlegm at all during the rest of the day or at night? | | | | | | | | | | | 1. Yes 2.No | | |  | | |  |
|  | | Do you bring up phlegm on most days for 3 consecutive months or more during the year? | | | | | | | | | | | 1. Yes 2.No | | |  | | |  |
|  | | For how many years have you had trouble with phlegm? | | | | | | | | | | | _____years | | |  | | |  |
| **C. Wheezing** | | | | | | | | | | | | | | | |  |  |  |  |
|  | | A1. Does your chest ever sound wheezy or whistling:  1. When you have a cold? | | | | | | | | | | | 1. Yes 2.No | | |  | | |  |
|  | | 2. Occasionally apart from colds? | | | | | | | | | | | 1. Yes 2.No | | |  | | |  |
|  | | 3. Most days or nights? | | | | | | | | | | | 1. Yes 2.No | | |  | | |  |
|  | | *IF yes to 1, 2, or 3 in A:* For how many years has this been present? | | | | | | | | | | | _____years | | |  | | |  |
|  | | A2. Have you ever had an ATTACK of wheezing that has made you feel short of breath? | | | | | | | | | | | 1. Yes 2.No | | |  | | |  |
|  | | *if yes to A2 in Q 38:* Have you ever required medicine or treatment for the(se) attack(s)? | | | | | | | | | | | 1. Yes 2.No | | |  | | |  |
| D.**Breathlessness** | | | | | | | | | | | | | | | |  |  |  |  |
|  | | Are you troubled by shortness of breath when hurrying on the level or walking up a slight hill? | | | | | | | | | | | 1. Yes 2.No | | |  | | |  |
|  | | Do you ever have to stop for breath when walking at your own pace on the level? | | | | | | | | | | | 1. Yes 2.No | | |  | | |  |
|  | | Do you ever have to stop for breath after walking about 100 yards (or after a few minutes) on the level? | | | | | | | | | | | 1. Yes 2.No | | |  | | |  |
|  | | Are you too breathless to leave the house or breathless on dressing or undressing? | | | | | | | | | | | 1. Yes 2.No | | |  | | |  |
|  |  | **2.diagnosis and other history** | | | | | | | | | | | | | |  |  |  |  |
|  | | Current diagnosis (reason of admission**)** | | | | | 1. Acute exacerbations of asthma  2. Acute exacerbations of COPD  3. ACOS  4.With other co morbidity (specify)________ | | | | | | | | |  | | |  |
|  | | Last hospitalizations( write specific month if exist) | | | | | | 1. >12 month  2. <12 month  3. others­­­_____ | | | | | | | |  | | |  |
|  | | Chest clinic physician visit | | | | | | 1. >12 month  2. <12 month  3. never visit | | | | | | | |  | | |  |
|  | | **Hospital readmission**  1.30 day readmission | 1. number of admissions *before and after index admission*,_______&________  2. Dates of index admission and discharge, ___________&__________  3. acute and rehabilitative lengths of stay in index admission,__________&___________  4. Date of first re-admission after discharge from index admission), ____________________  5.Number/frequency of readmission__________________________________ | | | | | | | | | | | | |  | | |  |
|  | | Do you have the following Co morbidity  Date of occur and Dx----------------- | 1.heart failure(IHD) 2.hypertension 3.diabetus M. 4.cardiac arrhythmia  5. Depression 6.pulmonary HTN 7. Renal failure 8. cancers  9. DVT 10. Others specify--------------------------------------------------- | | | | | | | | | | | | |  | | |  |
|  | | investivagation**s**  Date of investigated  ----------------------- | - hemoglobin, =______________spo_2=________________________________ - total white blood cell count,=_________________ - creatinine=______________ - albumin=______________ - urea =_________________ - BUN=_________________ | | | | | | | | | | | | |  | | |  |
| **Lung functions test (date and time -----------------** | | | | | | | | | | | | | | | | |  |  |  |
|  | a.Pre bronchodilator spirometry | | | | 1. FVC=______________________________  2. FVC, % predicted=___________________PEF=-------------------------  3. FEV 1 =________________  4. FEV 1 , % predicted =________________  5. FEV 1 /FVC =____________________ | | | | | | | | | | | |  | | |
|  | **b**.Postbronchodilator spirometry | | | | 1. FVC=______________________________  2. FVC, % predicted=___________________PEF=--------------------------  3. FEV 1 =________________  4. FEV 1 , % predicted =________________  5. FEV 1 /FVC =____________________ | | | | | | | | | | | |  | | |
|  | Severity GOLD/GINA scale | | | | 1. Moderate2. Severe 3. Very severe ,  spirometry **comment**________________ | | | | | | | | | | | |  | | |
|  | **a**. Past treatment and discharged medications | | | | 1. antibiotics 2. long-term oxygen therapy, 3. High dose inhaled SABA/ LABA/ ICS or systemic corticosteroid). 4. Baseline PaO2 values 5. Others (specify)________________________ | | | | | | | | | | | |  | | |
|  | **b**. Current treatment | | | | 1. Antibiotics ( Ceftriaxone, azithromycine, vancomycin,Doxycycline,Amox,others=--------------------------------- 2. Oxygen therapy 3. SABA (Salbutamol inhalation/tablet) 4. LABA (selmaterol inhalation, formoterolinhation) 5. ICS ( bechlomethasone inhalation 6. Systemic corticosteroids (Prednisolone, hydrocortisone inj, Dexamethasone inj, triamcinolone… 7. other combinations (budesonide with formoterol inhalations) | | | | | | | | | | | |  | | |
| **3. outcomes** | | | | | | | | | | | | | | | | |  |  |  |
| 1. **outcomes** | | | | types | | | | | | Date of Dx | Rx given | | | Method of diagnosis | | |  |  |  |
| Complications  1.In hospital  2.at admission | | | | 1.cor-pulmonale,  2.polyceythemia,  3.recurrent pneumonia,  4. Pneumothorax,  5. depression, 6.anemia, 7.respiratory failure,  8.atelectasis | | | | | |  |  | | | 1.Lab.Investigations  2. Physician summary note on chart. | | |  |  |  |
| 2.deteriorate | | | | **possible evidence of deterioration**   1. decrease BMI 2. .Organ failure 3. hypoxia 4. others----------------- | | | | | |  |  | | |  | | |  |  |  |
| 3. improved | | | | 1. Yes 2. NO | | | | | |  |  | | |  | | |  |  |  |
| . 4.LOS_______________day/month | | | | Date admitted-----------------------  Date discharged--------------- | | | | | |  |  | | |  | | |  |  |  |
| 5. mortality (death)—a. in-hospital b. after discharge | | | |  | | | | | |  |  | | |  | | |  |  |  |

1. **Checking Adherence Errors in inhaler technique in COPD and asthma groups**

Instructions for MDI *or* Aerolizer *or* Turbuhaler *or* Diskus **( a. asthma, b. COPD)**

1. Adherence to medication inhalation techniques after all? **1.yes 2.No**

| ***Is the patients able to know to----?????*** | Correct use of the techniques |
| --- | --- |
| 1.Take off the inhaler cap | 1. Yes 2. No |
| 2.Shake the MDI before use | 1. Yes 2. No |
| 3.Hold the MDI in a vertical position | 1. Yes 2. No |
| 4.Hold your head in a vertical position | 1. Yes 2. No |
| 5.Exhale before use | 1. Yes 2. No |
| 6.Put the mouthpiece in your mouth, and close your lips | 1. Yes 2. No |
| 7.Press the canister when inhaling slowly | 1. Yes 2. No |
| 8.Inhale deeply | 1. Yes 2. No |
| 9.Hold your breath for 10 seconds | 1. Yes 2. No |
| 10.Exhale and wait for 30–60 seconds before the other puff | 1. Yes 2. No |

NB: pMDI : Slow and Steady,DPI: Quick and Deep, MDI=metered dose inhalations, DPI= dry powder inhalations.
