# Supplementary material for: Early readmission and its predictors among patients treated for acute exacerbations of chronic obstructive respiratory disease in Ethiopia: A prospective cohort study
Source: PLoS One. 2020 Oct 6;15(10):e0239665. doi: 10.1371/journal.pone.0239665 (PMC7537865; doi:10.1371/journal.pone.0239665)
Supplement: S2 File — (DOCX) [file pone.0239665.s002.docx]

ጅማ ዩኒቨርሲቲ

ጤ ና ሳይንስ እንስቲቱት

ፈርማ ሲ ትም ርት ክፍል

**Participant Written Informed Consent Form: Amharic Version**

**የስም ም ት ሰነድ**

**ዉ ድ የጥናቱ ተሳተፊዎች**

አቶ ዘነበ ቀኖ በፋርማ ሲ ት /ት ክፍል የማ ስተርስ ድግሪ የመመ ረቅያ ምርምሩን በዚህ ሆስፒታል ለህክምና በተኙ ታካሚዎች ለይ ስለ የተባበሰ አስም እና የቆየ የሰንባ ስርሰደድበ ሽታና ተባብሶበ ቀን ዉስጥ ተመልሶየመተኛትእንድሁምምክናተቸዉስምንእንደሆነየሚያጠና፤ሲሆን የጥናቱ ዋና አላማ መ ረጃ ተኮር ስለየተባበሰ አስም እና የቆየ የሰንባ ስር ሰደድ በሽታ ናተባብሶበ ቀን ዉስጥ ተመልሶ የመተኛት እንድሁም ምክንያናተቸዉስ ምን እንደሆነማቅረብ፤ ለድሪጅቱም ሆነ ለጤና በለሞያ እጅግ በጣም አስፈላጊ ይሆናል።ይህ ጥናት አስፈላጊነቱ በዋናነት እንደዚህ አይነት ችግሮች ወደ ፊት ለመከላከል የሚያስችል ስልትለመቀየስየሚጠቅምነው፡፡

የእናንተ ተሳትፎ በዚህ ምርምር ላይ በፍቃደኝነት ላይ የተመሰረተ ሲሆን በማንኛውም ሰዓት በምትፈልጉበት ግዜ ከምርምሩ ራሳችሁን ማግለል ትችላላችሁ፡፡ስለ እናንተ ማንነት የሚገልጹ መረጃዎች ጥናቱ በሚስጥር የሚይዝ ሲሆን መረጃዎችንም ለሌላ ሶስተኛ ወገን አሳልፎ አይሰጥም፡፡

በዚህ ምርምር ላይ በመሳተፍ በቀጥታ የሚያስገኝልዎት ጥቅም ባይኖርም ምርምሩ በርስዎ ላይ ምንም አይነት ጉዳት አያደርስም፡፡በጥናቱ ላይ ያለዎትን ጥያቄ ለአቶ ዘነበ ቀኖ በስልክ ቁጥር 0912812388 ወይም Email: [zenebekano21@gmail.com](mailto:zenebekano21@gmail.com)ማ ስተላለፍ እንደሚ ችሉ እየገለጽኩ ስለትብብርዎ እናመሰግናለን፡፡

ቀን______________________

የጠ ያቂው ፊርማ______________

የጥናቱ ተሳታፊ ፊርማ___________

## Data collection format in Amharic version

| No. | ጥያቄዎች | | | | ምሊሾች | | | | | | ምርመራ |
| --- | --- | --- | --- | --- | --- | --- | --- | --- | --- | --- | --- |
|  | ፆታ | | | | 1. ወንድ 2. ሴት  **3. ስልክ ቁጥር=**__________________________ | | | | | |  |
|  | ዕድሜ | | | | 1.______ዓመት 2. ክብደት(kg)______  3. ሪዝመት_____ሜ 4. የሰዉነት አመካይ ክብደት _________ | | | | | |  |
|  | የጋብቻ ሁኔታ | | | | 1. ያለገባ/ች 2. ያገባ/ች 3. አግብቶ/ታ የፈታ/ች 4. የትዲር አጋር በሞት ያጣ/ች | | | | | |  |
|  | በቋሚነት የሚኖሩበት ቦታ የትነው? | | | | 1. ከተማ 2. ገጠር | | | | | |  |
|  | የትምህርት ዯረጃ | | | | 1. ያሌተማረ/ች 2.የመጀመሪያ ዯረጃ 3. ሁሇተኛ ዯረጃ 4. ኮላጅ ወይም ዩኒቨርሲቲ | | | | | |  |
|  | ስራ | | | | 1. የመንግስት ሰራተኛ 2.የግሌ ተቀጣሪ 3. ነጋዴ 4. አርሶ አደር 5. የቤት እመቤት 6. የቀን ሰራተኛ 7.የእንዱስትሪ ሰረተኛ 8. ሌላ ካሆነ ይጥቀሱ …………… | | | | | |  |
|  | ወርሃዊ ገቢዎ ምን ያህሌ ነው? | | | | _______________________ ኢቲዮጵያ ብር | | | | | |  |
|  | የቤት ሁኔታ | | | | 1. ከቤተሰበ ጋር ነዉ 2. ብቻ ነዉ | | | | | |  |
|  | ከቤት እንሰሳ ጋር ይኖራሉ | | | | 1. አዎ 2. አይደለም | | | | | |  |
|  | የመጨሽ ሁኔታ | | | | 1. አጫሽ ነዉ 2. አጫሽ አይደለም 3. አጫሽ አይደለም ገን ተጋለጭ ነዉ | | | | | |  |
|  | አጫሽ ከሆኑ ለQ10 | | | | 1.______ፓኮ/በቀን(ግምታዊ)  2. ______ፓኮ/በወር(ግምታዊ)  3. ______ፓኮ/በዓመት(ግምታዊ) | | | | | |  |
| ክፍል2: የህክምና ታሪክ | | | | | | | | | | | |
|  | | የመተንፈሻ ቧንባ በሽታ ምን ያክል ግዜ ሆኗል | | 1. ስር ሰደድ በሽታ_______ወር/ዓመት  2. አስማ________ ወር/ዓመት  3. የሁለቱ ጥምር_________________ ወር/ዓመት | | | | |  | | |
|  | | ሆስፕታል ተኝቶ ያቃሉ? | | 1. አዎ 2. አይደለም | | | | |  | | |
|  | | መልስዎት አዎ ከሆነ ለQ13, | | የተኙበት ዋና ምኪኒያት: 1. ለ አስማ  2. ለስር ሰደድ በሽታ3. ለሁለቱ ጥምር 4. ሌላ ከአለ ይግለፁ____________ | | | | |  | | |
|  | | መልስዎት አዎ ከሆነ ለQ13, ለምን የህል ግዜ ነዉ | | ­­­­­­­­­­­­­­­­­­_________ቀን or___________ወር/ዓመት | | | | |  | | |
|  | | በጸና የታመ ሙ ሰወች ከሚ ታከሙ በት(ICU) ክፍሌልገብተው ታክመ ው ያው ቃሉ? | | 1. አዎ 2. አይደለም | | | | |  | | |
|  | | በመ ሳሪያ የታገዘ አየር ተሰጦወት ያውቃል | | 1. አዎ 2. አይደለም | | | | |  | | |
|  | | መልስዎት አዎ ከሆነ ለQ17 | | በቀን ምን ያክል ኦከሲጂን ለይ ይቆያሉ_______(ግምታዊ in hrs/24hours | | | | |  | | |
|  | | እንቅልፍ እመቢ ይሌወታሌ | | 1. አዎ 2. አይደለም | | | | |  | | |
|  | | መልስዎት አዎ ከሆነ ለQ18 | | በለሊት ዉስጥ ምን ያህል ያስቻግራል_______ ( ይጥቀሱ) | | | | |  | | |
|  | | ያለ ረደት የእለት ከእለት የቤት ውስጥ ሰራዎትን መስራት ይችሊል(ትንፊሽ ማጠሩ ስራወትን እንዲይሰሩ  ከልክሎወታል ) | | 1. አዎ 2. አይደለም | | | | |  | | |
| ክፍል III-የክሊኒክ ባህርያት እና ተያያዥ ነገሮች  1. ክሊኒካዊ አቀራረቦች (ምልክቶች እና ምልክቶች)(**Clinical presentations(sign and symptoms)**  **ሀ. ሳል በተመለከተ** | | | | | | | | | | | |
|  | አብዛኛውን ጊዜ ሳልዎ ነው? (በመጀመሪያ ጢስ ወይም በቤትመውጣት ሲጀምሩ ያስቁሙ) | | | | | | | 1. አዎ 2.አየደለመ | |  | |
|  | በአብዛኛው በቀን ከ 4 እስከ 6 ጊዜያት ይሳለሉ, በሳምንቱ ውስጥ 4 ወይም ከዚያ በላይ ቀናት ይሳላሉ? | | | | | | | 1. አዎ 2.አየደለመ | |  | |
|  | አብዛኛውን ጊዜ ከመነሳትዎ በፊት ወይም በጧት ላይ ይሳለሉ? | | | | | | | 1. አዎ 2. አይደለም. | |  | |
|  | በአብዛኛው ቀኑን ሙሉ ወይምማታ ያስሎታል? | | | | | | | 1. አዎ 2.አይደለመ | |  | |
|  | ብዙጊዜ ለ 5 ተከታታይወራት ወይም ከዚያ በላይ ብዙይሳላሉ? | | | | | | | 1. አዎ 2.አይደለመ | |  | |
| **ለ. Phlegm(አክታ)** | | | | | | | | | | | |
|  | ብዙውን ጊዜ ከደረትዎ ላይ ነጠብጣብ ይዘው ይወጣሉ? (ከመጀመሪያው ጭስ ጋር ወይም ከቤት ውጪ ሲወጡ የመጀመሪያ ቁጥር አክታዉ ወይስ ከቤት? | | | | | | | 1. አዎ 2.አይደለመ | |  | |
|  | ብዙውን ጊዜ በቀን ሁለት ጊዜ እንደዚህ ዓይነቱ አክታ ያመጣሉ, ከሳምንቱ አራት ቀን ወይም ከዚያ በላይ ቀናት ያወጡልዎታል? | | | | | | | 1. አዎ 2.አይደለመ | |  | |
|  | ብዙውን ጊዜ በማለዳ ላይ ወይም የመጀመሪያውን ስራ ለመጀመር ብዙጊዜ አክታዎን ያመጣሉ ወይ? | | | | | | | 1. አዎ 2.አይደለመ | |  | |
|  | በአብዛኛው ቀኑን በቀን ወይም ማታ ላይ ነጠብጣብዎን ያመጣሉ ወይ? | | | | | | | 1. አዎ 2.አይደለመ | |  | |
|  | በአመቱ ውስጥ ለ 3 ተከታታይ ወራት ወይም ከዚያ በላይ በአብዛኛዎቹ ቀናት ውስጥ አክታዉን ከፍያደርጉዎታል? | | | | | | | 1. አዎ 2.አይደለመ | |  | |
|  | ለምን ያህል አመታት በአክታ ችግርአጋጥሞዎታል? | | | | | | | ________አመት | |  | |
| **C. Wheezing(የመተንፈስ ችግር)** | | | | | | | | | | | |
|  | A1. ደረተዎ ለይ የመተንፈስ ችግር ኣለ፡ 1. በቀዝቃዛ ግዜ? | | | | | | | 1. አዎ 2.አይደለመ | |  | |
|  | 2. አንድ አንዴ ከቀዝቃዛ ቦታ ከራቁስ? | | | | | | | 1. አዎ 2.አይደለመ | |  | |
|  | 3. አብዛኛዉ ቀን ወይስ ማታ ማታ? | | | | | | | 1. አዎ 2.አይደለመ | |  | |
|  | *ለ1, 2, ወይም 3 መልሰዎት አዎ ከሆነ ሀ1:* ለምን ያህል ግዜ ነበረቦዎት? | | | | | | | _____አመት | |  | |
|  | ሀ2. አተነፋፈስ እንዲ ሰማዎት ያደርግዎት የነበረውን የትንፋሽ ብርታትን አግኝተው ያውቃል? | | | | | | | 1. አዎ 2.አይደለመ | |  | |
|  | *ለ ሀ2 መልስዎት አዎ ከሆነ በ ቁጥር 35ለይ:* ለተሰመዉ ትጥቃት መድሃኒት ወይም ህክምና ጠይቀዋል? | | | | | | | 1. አዎ 2.አይደለመ | |  | |
| D. **Breathlessness** (**እስትንፋስ የመጠር)** | | | | | | | | | | | |
|  | በደረጃ ላይ በፍጥነት በመሄድ ወይም በትንሽ ኮረብ ላይ ሲራመዱ በትንሽ ትንፋሹ ይረበሻል? | | | | | | | 1. አዎ 2.አይደለመ | |  | |
|  | በየደረጃዎ በእራስ ፍጥነት ሲጓዙ ለትንፋሽ መቆም ያስፈልግዎታል? | | | | | | | 1. አዎ 2.አይደለመ | |  | |
|  | 100 ያረድስ (ከትንሽ ደቂቃ ቧሃላ) ለ ማቆም ያስፋልጎዎታል? | | | | | | | 1. አዎ 2.አይደለመ | |  | |
|  | ከቤት ወጥቶ ለመልበስ አልያም በአለባበስ ለይ የመተንፈስ ችግር ያጋጥማል? | | | | | | | 1. አዎ 2.አይደለመ | |  | |
|  | **2.diagnosis and other history** | | | | | | | | | | |
|  | Current diagnosis (reason of admission**)** | | | | | 1. Acute exacerbations of asthma  2. Acute exacerbations of COPD  3. ACOS  4.With other co morbidity (specify)_________ | | | |  | |
|  | Last hospitalizations( write specific month if exist) | | | | | | 1. >12 month  2. <12 month  3. others­­­_____ | | |  | |
|  |  | | | | | |  | | |  | |
|  | Chest clinic physician visit | | | | | | 1. >12 month  2. <12 month  3. never visit | | |  | |
|  | Hospital readmission | | 1. number of admissions before and after index admission, _________&______________  2. Dates of index admission and discharge, _________________&__________________  3. acute and rehabilitative lengths of stay in index admission,__________&___________  4. Date of first re-admission after discharge from index admission), ____________________ | | | | | | |  | |
|  | Co morbidity | | **1.____________________**  **2._______________________**  **3._______________________**  **4._______________________**  **5.______________________** | | | | | | |  | |
|  | investivagation**s**  ABG analysis(if exist) | | - PH_______ - P co 2 ___________mm Hg - P o 2 ____________ mm Hg - actual bicarbonate=____________ - hemoglobin, =______________ - total white blood cell count,=___________ - creatinine=______________ - albumin=______________ | | | | | | |  | |
| **Lung functions test** | | | | | | | | | | | |
| 1. 47. | a.Pre bronchodilator spirometry | | 1. FVC=______________________________  2. FVC, % predicted=___________________  3. FEV 1 =________________  4. FEV 1 , % predicted =________________  5. FEV 1 /FVC =____________________ | | | | | | |  | |
| 1. 48. | **b**.Postbronchodilator spirometry | | 1. FVC=______________________________  2. FVC, % predicted=___________________  3. FEV 1 =________________  4. FEV 1 , % predicted =________________  5. FEV 1 /FVC =____________________ | | | | | | |  | |
|  | Severity GOLD/GINA scale | | 1.Moderate  2. Severe  3. Very severe | | | | | | |  | |
| **Treatment** | | | | | | | | | | | |
| 1. 50. | **a**. Past treatment and discharged medications | | 1. antibiotics 2. long-term oxygen therapy, 3. High dose inhaled or systemic corticosteroid). 4. Baseline PaO2 values 5. Others (specify)________________________ | | | | | | |  | |
| 1. 54 | **b**. Current treatment | | 1. antibiotics 2. Oxygen therapy 3. SABA 4. LABA 5. ICS 6. Systemic corticosteroids g. Or with combinations | | | | | | |  | |
| 1. 55 | outcomes | | **1.** complications(morbidity )---a. before admission b. in-hospital(after admission)  2.deteriorate  3. improvement  4. length of hospital stay_______________day/month  5. mortality (death)—a. in-hospital b. after discharge | | | | | | |  | |
